# Bacteria and Fungi Respond Differently to Multifactorial Climate Change in a Temperate Heathland, Traced with ^13^C-Glycine and FACE CO_2_

**DOI:** 10.1371/journal.pone.0085070

**Published:** 2014-01-15

**Authors:** Louise C. Andresen, Jennifer A. J. Dungait, Roland Bol, Merete B. Selsted, Per Ambus, Anders Michelsen

**Affiliations:** 1 Department of Biology, University of Copenhagen, Copenhagen, Denmark; 2 Sustainable Soils and Grassland Systems Department, Rothamsted Research-North Wyke, Okehampton, United Kingdom; 3 Institute of Bio- and Geosciences, Forschungszentrum Jülich, Jülich, Germany; 4 Chemical and Biochemical Engineering, Technical University of Denmark, Kongens Lyngby, Denmark; Wageningen University, Netherlands

## Abstract

It is vital to understand responses of soil microorganisms to predicted climate changes, as these directly control soil carbon (C) dynamics. The rate of turnover of soil organic carbon is mediated by soil microorganisms whose activity may be affected by climate change. After one year of multifactorial climate change treatments, at an undisturbed temperate heathland, soil microbial community dynamics were investigated by injection of a very small concentration (5.12 µg C g^−1^ soil) of ^13^C-labeled glycine (^13^C_2_, 99 atom %) to soils *in situ*. Plots were treated with elevated temperature (+1°C, T), summer drought (D) and elevated atmospheric carbon dioxide (510 ppm [CO2]), as well as combined treatments (TD, TCO2, DCO2 and TDCO2). The ^13^C enrichment of respired CO_2_ and of phospholipid fatty acids (PLFAs) was determined after 24 h. ^13^C-glycine incorporation into the biomarker PLFAs for specific microbial groups (Gram positive bacteria, Gram negative bacteria, actinobacteria and fungi) was quantified using gas chromatography-combustion-stable isotope ratio mass spectrometry (GC-C-IRMS).

Gram positive bacteria opportunistically utilized the freshly added glycine substrate, *i.e.* incorporated ^13^C in all treatments, whereas fungi had minor or no glycine derived ^13^C-enrichment, hence slowly reacting to a new substrate. The effects of elevated CO_2_ did suggest increased direct incorporation of glycine in microbial biomass, in particular in G^+^ bacteria, in an ecosystem subjected to elevated CO_2_. Warming decreased the concentration of PLFAs in general. The FACE CO_2_ was ^13^C-depleted (δ^13^C = 12.2‰) compared to ambient (δ^13^C = ∼−8‰), and this enabled observation of the integrated longer term responses of soil microorganisms to the FACE over one year. All together, the bacterial (and not fungal) utilization of glycine indicates substrate preference and resource partitioning in the microbial community, and therefore suggests a diversified response pattern to future changes in substrate availability and climatic factors.

## Introduction

Soils act as potential sinks or sources for atmospheric carbon dioxide (CO_2_) depending on the balance between carbon (C) inputs from net primary production (NPP) and the C efflux by autotrophic and heterotrophic respiration [1]. The organomineral soils of heathlands in the Northern Hemisphere are essential for global C storage and it is vital to understand whether these ecosystems will be a net sink of C under predicted climate change scenarios. Their soils are characteristically of low pH (<5) and of low nutrient availability because of the nutrient poor litter produced by the plant ecotypes, and the positive feedback from symbiotic ericoid and ectomycorrhizal fungi which are highly developed to scavenge for labile nutrient sources [2]. A switch from fungal-to-bacterial dominance in these systems caused by climate change could trigger the release of vast amounts of C from the terrestrial pool. Microbial utilization of ‘old’ or ‘new’ carbon (C) from soil organic matter (SOM) may cause a release of C from previously sequestered C stocks (i.e. ‘priming’ [3], [4]) or from recently assimilated CO_2_ [5], which influences the stabilization of C in soils [6], [7].

The quantity and quality of the organic matter (SOM) in soils may be strongly influenced by the combination of changes in atmospheric CO_2_ concentration, air and soil temperature and moisture regime, which directly affects NPP [1], [8]–[10], and is additionally regulated via other ecosystem processes, such as nutrient availability and herbivore abundance [11], [12]. In C3 plants in temperate heathlands, CO_2_ assimilation can be stimulated by elevated concentrations of CO_2_ [13]–[15], but if plant metabolism is limited by other factors such as nutrient availability, stores of carbohydrates may accumulate leading to a total higher tissue C to nitrogen (N) ratio [16]. The relatively smaller N content partially persists through wilting, but along with alteration of lipids (e.g. carboxylic acids; [17]), and suberin and cutin concentrations [18], the litter input to SOM may be of slightly poorer quality for decomposing organisms. In addition, elevated CO_2_ stimulates the efflux of labile organic compounds from roots into the soil, which diffuse passively due to the concentration gradient [19]. In plants under elevated CO_2_, glycine may be routed into the rhizosphere [20] due to changes in the citric-acid cycle caused by low nutrient availability. Overall, elevated CO_2_ may modify SOM quality through altered plant litter deposition and root exudation [21], while by contrast microbial residues in the SOM may not be affected [22]. Changes in the magnitude and chemistry of plant inputs to soils under climate change is, therefore, likely to affect the structure and function of the soil microbial community which drive the turnover of soil organic matter [23].

The net turnover of soil organic carbon in fungi dominated systems is slower than in bacterially dominated soils [24]. Only some fungi are able to complex organic polymers of the plant cell wall (i.e. lignocellulose), to access to access energy and nutrients, whilst bacteria generally rely on the availability of soluble substrates. Fungi also produce structures (i.e. chitin) that are relatively less available for decomposition [25] and have a higher C∶N ratio (5 to 15) compared with bacteria (3 to 6; [26]). Moreover, the ramification of fungal hyphae through soils encourage soil aggregate formation and thereby physical protection of organic C from decomposition [27]. In *Calluna vulgaris* dominated ecosystems the ericoid mycorrhizal fungus *Hymenoscyphus ericae* is associated with heather roots and transfers nutrients (N and phosphorus) to the host plant in return for labile carbohydrates [2]. The mycorrhizal fungi additionally use litter as a substrate through the release of exo-enzymes [28]. In contrast, soil bacteria grow on carbohydrate substrates in the soil. Experimental addition of sugars to the soil generally increases soil respiration [29] leading to enhanced bacterial growth and substrate assimilation [30], [31], while most fungal groups respond more slowly to addition of labile C [32]. This diversified utilization of substrates by different microbial groups suggests that changes in substrate input to the soil caused by changes in environmental drivers, may lead to altered composition of SOM [23]. This may have consequences for the wider soil food web because of the division of energy routed through fast bacterial or slow fungal channels [33].

Compound-specific stable isotope techniques have proven highly sensitive for detection of the selective incorporation of small concentrations of ^13^C-labelled compounds into specific microbial phospholipid fatty acid (PLFA) biomarkers, and is an established method for assessment of soil microbial populations after applications of ^13^C-labelled substrates [34], [35], or of ^13^C-depleted CO_2_ [7], [36], [37]. Recent compound specific studies of substrate incorporation into fungal and bacterial PLFA biomarkers have revealed substrate specificity between microbial groups, i.e. with differences in the incorporation of organic compounds as glucose [30], amino acids [38], , glycine [40], thymidine and leucine [31], [41], [42] or plant litter [43].

The effect of fluctuations in temperature and soil moisture content on soil microbial activity have been explored in many soils (e.g. [44], [45]), and elevated CO_2_ also causes shifts in soil microbial community compositions which is observed under FACE conditions (e.g. [23], [46]) or in plant-free soil incubations [47]. However, the opportunity to explore the microbiology of soils under combinations of predicted climate change variables under field conditions is rare [48], [49].

During three years of manipulation treatment with elevated CO_2_, warming and summer drought at the experimental heathland site CLIMAITE [50], a consistent c. 30% increase in soil respiration was observed in response to elevated CO_2_, and drought periodically reduced soil respiration [51]. Here, we attempt to explore the underlying mechanisms causing a possible shift in substrate utilization by bacterial and fungal groups, by identifying the group of soil microorganisms that respond to changes in availability of labile metabolites in root exudates. We explore the effect of controlled climate change manipulations at field scale on microbial community structure and diversity responses, and the effects of increased availability of glycine using changes in biomarker PLFA abundance and ^13^C incorporation. Two ^13^C tracing approaches were used to investigate the activity of soil microorganisms under different climate change conditions: (i) rapid response (24 h) of the microbial biomass to addition of the elicitor ^13^C-labelled glycine to the soil and (ii) longer term microbial dynamics (one year) using indirect labeling via ^13^C-depletion of the plant biomass and plant litter obtained by FACE fumigation. Our hypotheses were: I. Treatments with warming decrease the abundance of fungal PLFA biomarker; II. FACE treatment (elevated CO_2_) primes SOM decomposition [52], and hereby increases bacterial glycine incorporation.

## Materials and Methods

### Site description

The field site was located in a public area owned by the Danish Government and under administration by the Danish Defence who granted permission to establish the experiment. The experiment took place at ‘CLIMAITE’ field site in Brandbjerg (55′53″N 11′58″E) c. 50 km NW of Copenhagen, Denmark. The site was a managed, dry, temperate heath on a hilly nutrient-poor sandy deposit, with an organic layer of c. 5 cm depth and a pH of 3.5 (in 0.01M CaCl_2_). The vegetation was dominated by *Calluna vulgaris*, *Deschampsia flexuosa* and heathland mosses and herbs. The average annual precipitation was about 600 mm and the average temperature was 8°C.

### Treatments

The climate manipulations at the ‘CLIMAITE’ field site started in 2005 [50] and consisted of eight treatments: elevated temperature (T), extended summer drought (D), elevated atmospheric CO_2_ concentration (CO_2_), all combinations of these treatments (TD, TCO_2_, DCO_2_ and TDCO_2_) and untreated reference plots (A), each in six replicates. The field site covered an area of about 2 ha and the experimental plots were distributed in 12 seven meter diameter octagons arranged pair-wise in six blocks. The temperature was increased by passive night-time warming, by means of automatic curtains. The drought treatment was imposed by using automatic curtains to remove precipitation. The atmospheric CO_2_ was increased by a regular FACE technique including feedback control on CO_2_ concentrations from wind speed and wind direction. The δ^13^C of CO_2_ in the ambient atmosphere and in the atmosphere with elevated CO_2_ concentration (FACE) were −8‰ and −12.2‰ respectively, averaged over the two years [53]. The temperature increase in 2 cm soil depth averaged 1°C, and the average CO_2_ concentration in the FACE plots was 510 ppm. The drought treatment excluded 54 mm summer rain in July 2006, equivalent to 9% of annual rainfall. For further information about the experimental design see [50].

### Isotope labeling

Dual-labeled glycine (^13^C_2_^15^N-glycine, 99% labeled on both C atoms and 99% labeled with ^15^N; Cambridge Isotope Laboratories) was added *in situ* in the end of September 2006 to 20 cm^2^ sub-plots of undisturbed vegetation, providing 0.1 L of re-demineralized water with 0.027 g glycine, corresponding to 687 mg glycine·m^−2^ (223 mg C·m^−2^ or 0.016 mg glycine·g^−1^ DW soil). The label was injected into the soil in 16 points of a grid fixed in a transparent plastic plate, just below the soil surface, by using a syringe connected to a dispenser on a 1 L glass bottle [54], [55]. The soil naturally contained free amino acids, the total total amino acid N of the free amino acids was 25.7±4.5 g N·m^−2^ in August 2005 [56].

Two types of ^13^C label were mixed during this experiment in the undisturbed heath: **1.** the highly enriched ^13^C label from glycine added to the soil in all plots and **2.** the slightly depleted ^13^C label from the FACE CO_2_ air in plots with elevated CO_2_, which was sourced through the plant and made available for microbes in the soil.

### Soil gas δ^13^C–CO_2_

Soil gas was sampled at 1 cm depth in control plots at intervals before and after glycine label addition. A 20 ml sample was extracted directly from the soil by hand from four points with a 25 mL plastic syringe and immediately flushed through the septum of a crimp sealed 1.8 ml vial. The vials were left over-pressurized with c. 1 ml sample until analysis of δ^13^CO_2_ at gas chromatograph (GC) (Hewlett–Packard 6890) in continuous flow mode with a Preparation Concentration unit (PreCon, Thermo Scientific, Germany) and stable isotope ratio mass spectrometer (IRMS) (Finnigan Delta PLUS, Thermo Scientific, Germany). These analyses were made at Technical University of Denmark.

### Soil sampling

Soil cores were sampled at the CLIMAITE field site from the soil surface (including the organic top-layer) and cut at 5 cm depth one day (c. 24 hrs.) after labeling with glycine. Three soil cores from each plot were mixed to a composite sample from each depth and immediately sorted into soil and roots by hand (no sieving). The samples were kept on ice until further processing. A subsample of the fresh soil from each plot was freeze dried for PLFA extractions.

Soil organic matter content (SOM) was determined by burning soil at 550°C for 6 h (loss on ignition) at University of Copenhagen.

Just before the labeling was performed, additional soil samples were taken in adjacent subplots within the climate treated plots to obtain δ^15^N and δ^13^C natural abundance signatures.

### Phospholipid fatty acid (PLFA) extraction

A modified Bligh Dyer extraction procedure was applied for the extraction of PLFA. Bligh Dyer solvent (potassium dihydrogen phosphate-buffered H_2_O at pH 7.2, methanol and chloroform in 4∶10∶5 *v∶v∶v*) was added to freeze-dried soil in a 3∶1 v∶w ratio, and ultrasonicated prior to centrifugation. The supernatant was removed and saved and the extraction procedure repeated a further three times. The supernatants were combined and chloroform and buffered water added to break the aqueous and organic phases. The organic phase was removed and saved and the aqueous phase extracted (three times) further with chloroform. All organic fractions were combined as a total lipid extract (TLE) and solvent evaporated under N_2_ atmosphere at 40°C. Silicic fractionation was used to isolate the phospholipid fraction from the TLE using the solvent system: chloroform (neutral lipids), acetone (glycolipids), methanol (polar lipids). Nonadecane in hexane was added to the polar fraction as the internal standard. After saponification (0.5 M methanolic NaOH; 1 h, 80°C) and acidification (1 M HCl), the liberated PLFAs were extracted with hexane, which was removed under N_2_ (40°C). Acidified dry methanol was added to methylate the PLFAs (1 hour; 80°C), which were extracted as their methyl esters with diethyl ether after cooling and addition of DCM extracted double distilled water. The DCM was evaporated under N_2_ (40°C), and the sample dissolved in hexane for GC analysis.

PLFA methyl esters (FAMEs) were quantified using a GC (Agilent 7890A, Agilent Technologies, Wokingham, UK) equipped with a DB-225 (30 m×25 mm (i.d.)×0.25 µm phase thickness). The temperature program was: 1 minute isothermal at 50°C, followed by a ramp to 150°C at 15°C minute^−1^, followed by an increase to 240°C at 4°C minute^−1^ and then held isothermally at 240°C for 20 minutes.

FAMEs were identified using GC-MS analyses, with GC conditions the same as above, except He was used as the carrier gas in all cases. The interface was set to the maximum oven temperature, the ion source was set to 30°C below this and the quadropole MS scanned the range of m/z 50–650 at 1.7 scans per s. Data were acquired and processed using the Chemstation data system.

### PLFA analysis GC and GC-C-IRMS

FAME samples were analyzed at The James Hutton Institute by GC-C-IRMS using a Trace GC Ultra gas chromatograph (Thermo Finnigan, Bremen, Germany) equipped with a GC PAL auto sampler (CTC Analytics AG, Zwingen, Switzerland) using the method described in [57]. The Trace GC Ultra contained an HP-5 column (50 m length, 0.2 mm i.d., film thickness of 0.33 µm; Agilent Technologies/J&W Scientific). The samples were injected in split-less mode via an inlet held at 250°C, and the He carrier gas was maintained at a constant flow rate of 1.5 mL min^−1^. The GC oven was initially set at 100°C, held for 1 min, ramped at 20°C min^−1^ to 190°C, then at 1.5°C min^−1^ to 235°C, and finally at 20°C min^−1^ to 295°C; where the temperature was held for 15 min. A GC Combustion interface III was used to link the GC to a Delta V Advantage IRMS (both Thermo Finnigan). The oxidation reactor on the interface was maintained at 950°C and the reduction reactor at 650°C. Isodat 3.0 Gas Isotope Ratio MS Software (version 3.0; ThermoFisher Scientific) was used for data processing. For each peak the combined area of the peaks for all the ions (*m/z* 44, 45 and 46) after background subtraction was collected.

### Calculations

Total bacteria were identified by the PLFAs: 15:0, 17:0, *i*15:0, *a*15:0, *i*17:0, *a*17:0, 10Me16:0, 10Me17:0, 10Me18:0, *cy*17:0, 18:1ω9c, *cy*19:0, and fungi were identified with the PLFA 18:2ω6,9c. Bacterial PLFAs were further grouped into gram positive (G^+^) bacteria (*i*15:0, *a*15:0, *i*17:0, *a*17:0, 10Me16:0, 10Me17:0, 10Me18:0), actinobacteria (10Me16:0, 10Me17:0, 10Me18:0), and gram negative (G^−^) bacteria (*cy*17:0, 18:1ω9c, *cy*19:0) [58], [59].

The sum of the weighted mean frequency (PLFA diversity: Shannon-Weaver index for information density [24], [61], [62]) was calculated as the sum of each individual frequency weighted by the logarithm of the frequency (P):
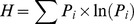
(I)Bacterial stress index (BSI) [60] was calculated as:
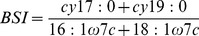
(II)The specific microbial activity (τ) calculated for each individual biomarker as:
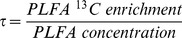
(III)


Due to the ^13^C-depleted labeling form the FACE air [1], [53] each PLFA had a different baseline (^13^C atom%) in ambient air plots compared to CO_2_ treated plots [63]. This was used for calculation of ^13^C enrichments for each biomarker individually. Hence the ^13^C baseline from ambient was used for A, T, D and TD plots while the ^13^C baseline from CO_2_ treated plots was used for CO_2_, TCO_2_, DCO_2_ and TDCO_2_. The δ^13^C of individual PLFA biomarkers ranged from 0.03 to 2.9‰ between A and CO_2_ treatment and was significantly different for the bacterial biomarkers: 15:0 (P = 0.0396) and *a*15:0 (P = 0.0472).

### Statistical methods

Linear mixed models (proc. mixed and GLM in SAS) were applied to analyze the effect of treatment on: PLFA concentrations and ^13^C-enrichment of PLFAs. Random effect terms were block, octagon and plot, respecting the nested structure of the design. Main effect terms were the treatment factors: CO_2_, temperature (T) and drought (D); in addition, all interaction terms between the factors CO_2_, T and D were included. Root biomass was taken in as co-variate in proc. mixed. The models were gradually simplified, starting with third order interactions, taking out non-significant interactions until only significant terms were left. Statistics were carried out using SAS® 9.1 [64] and for all tests, a significance level of 0.05 was applied.

## Results

### Effect of climate change on soil community dynamics

**Figure 1:**
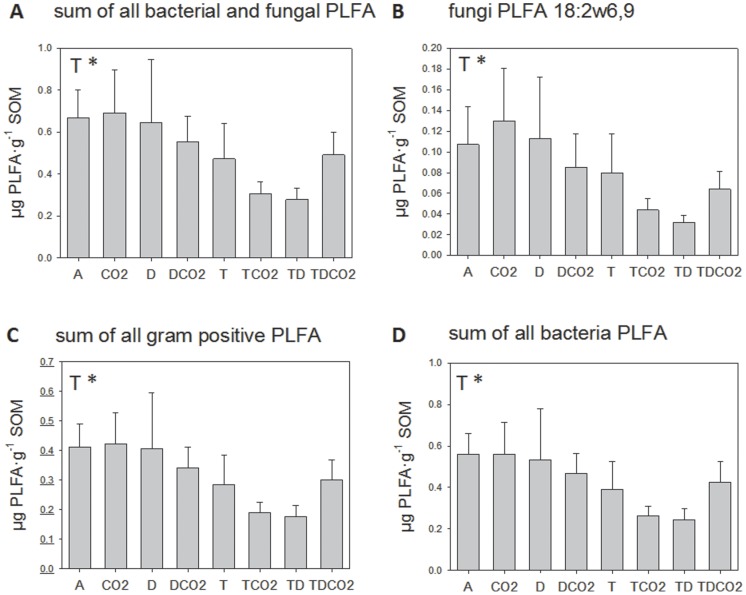
Concentration (µg PLFA·g^−1^ SOM) in top soil of PLFAs. Sum of all bacterial and fungal PLFA biomarkers (**A**); the fungal PLFA biomarker 18:2ω6,9 (**B**); all gram positive biomarkers (**C**) and all bacteria PLFA biomarkers (**D**); one day after addition of glycine to field plots subjected to *in situ* treatment through one year with the climate change factors: elevated atmospheric CO_2_ concentration (CO2), summer drought (D) and warming (T), and those treatments in all combinations. A is ambient control = no treatment. Error bars represent standard error. Significant effects of treatments: * is P<0.05.

The sum of the concentrations of the biomarker PLFA (Σ PLFA) was used to estimate differences in the size of the soil microbial community under each climate treatment, relative to the control plots (A):1.0(CO2), 1.0(D), 0.8(DCO2), 0.7(T), 0.5(TCO2), 0.4(TD), and 0.7(TDCO2). The warming treatments reduced Σ PLFA overall (Table 1 and Figure 1a) and the effect was similar for fungi (Figure 1b) and different bacterial groups (Figure 1 c and d). The sum of the weighted mean frequency (Equation I) of the bacterial and fungal PLFA biomarkers was significantly reduced by warming (P = 0.0495 for T) (*e.g.* index was H = 1.03±0.19 in A (ambient, no treatment) and H = 0.787±0.15 in T plots). There was no effect of treatment on BSI (Equation II; 7.0 in A (ambient, no treatment) and 8.5 in the CO_2_ treatment) or on the PLFA biomarker concentrations before addition of glycine.

**Table 1:** Significant effect of climate change treatments (P-values) on PLFA ^13^C enrichment from soils from the CLIMAITE experiment sampled in August 2007.

PLFA	Treatment effect	Biomarker
	P	Type
Σ(bacteria and fungi)	T: 0.0296	Σ(bacteria and fungi)
Σ(gram positive bacteria)	T: 0.0231	Σ(gram positive bacteria)
Σ(bacteria)	T: 0.028	Σ(bacteria)
Σ(actino bacteria)	T: 0.0267	Σ(actino bacteria)
18:2ω6,9	T: 0.0477	Fungi
*i*15:0	T: 0.0371	gram^+^ bacteria
*a*15:0	T: 0.0059	gram^+^ bacteria
*i*16:0	D*CO_2_: 0.0464	non-specific
10Me16:0	T: 0.0272	gram^+^ bacteria (actino)
*i*17:0	T: 0.0232	gram^+^ bacteria
17:0	T: 0.0479	Bacteria
10Me17:0	T: 0.0264	gram^+^ bacteria (actino)
18:0	T: 0.0386	non-specific
18:1ω9	T: 0.0361	gram^−^ bacteria

Soil was sampled one day after addition of ^13^C_2_-glycine to field plots subjected to *in situ* treatment through one year with the climate change factors: elevated atmospheric CO_2_ concentration (CO2) summer drought (D) and warming (T), and those treatments in all combinations. Linear mixed models (proc mixed and GLM in SAS) with main effects: CO_2_, temperature (T) and drought (D) and interactions between the factors CO_2_, T and D.

### Effect of glycine addition on soil microbial community structure

The ratio of total bacterial to fungal biomarkers was 7.5 (±1.8) in A (ambient, no treatment) after glycine addition, with no significant effect of treatment or addition of glycine. The ratio of actinobacterial to G^−^ bacterial biomarkers was 3.8 (±0.1; in A) after glycine addition and that of all G^+^ to G^−^ biomarkers was 5.4 (±0.1; in A) after glycine addition, and both increased as effect of glycine addition for A and CO_2_ treatments (P = 0.0002 and 0.0056, respectively). In response to CO_2_ treatments, actinobacteria to G− bacteria ratio (proc. mixed; CO_2_: 0.0663) and all G^+^ to G^−^ bacteria ratio (proc. mixed; CO_2_: 0.0581 and T: 0.0998) tended to increase after glycine addition, but not in treatments including warming treatment.

### Effect of climate change on incorporation of ^13^C from glycine

**Figure 2:**
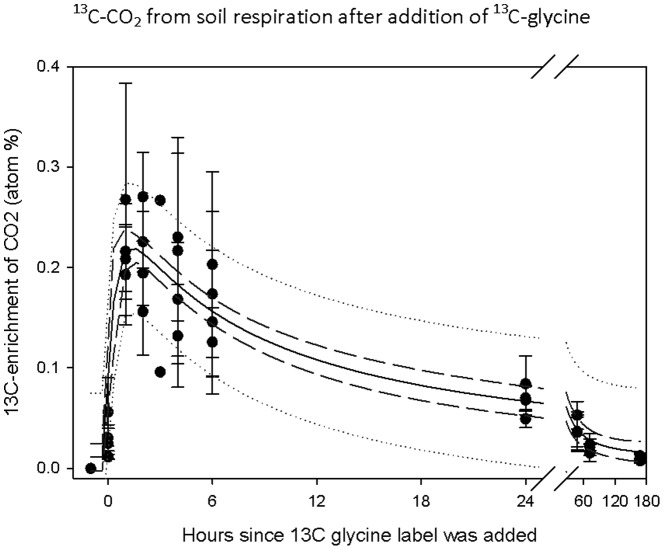
^13^C-CO_2_ enrichment in soil air (atom %). A time-series beginning a few hours prior to addition of the ^13^C-enriched glycine label till 168 hours after addition fit a log-normal curve: ^13^C-CO_2_ = 1.108+1.359*exp(−0.5*(ln(T/3.269)/1.797)^2^)/T); (R^2^ = 0.8832). Curve indicates average, 95% Confidence Band and 95% Prediction Band.

The ^13^C-CO_2_ enrichment in soil air measured in a time-series beginning a few hours prior to addition of the ^13^C-enriched glycine label till 168 hours after addition, fit a log-normal curve: ^13^C-CO_2_ = 1.108+1.359·exp(−0.5·(ln(T/3.269)/1.797)^2^)/T) (R^2^ = 0.8832, Figure 2). This indicates microbial respiration during glycine acquisition with maximum microbial activity before 6 h, and this had declined by 70% after 24 hours. This indicated active up take of the ^13^C-label within the timeframe of the experiment. Thereafter, the ^13^C from glycine was respired at a stable rate through the next week (Figure 2). There was no significant effect of treatment on the ^13^C-CO_2_ enrichment.

The addition of ^13^C_2_^15^N labeled glycine resulted in substantial microbial uptake during the first 24 hrs. The overall microbial enrichment with ^15^N and ^13^C correlated significantly in 0–5 cm depth, with ^13^C = 1.74·^15^N (R^2^ = 0.92, P<0.0001; [55], corresponding to N uptake with 87% in form of intact glycine.

**Figure 3:**
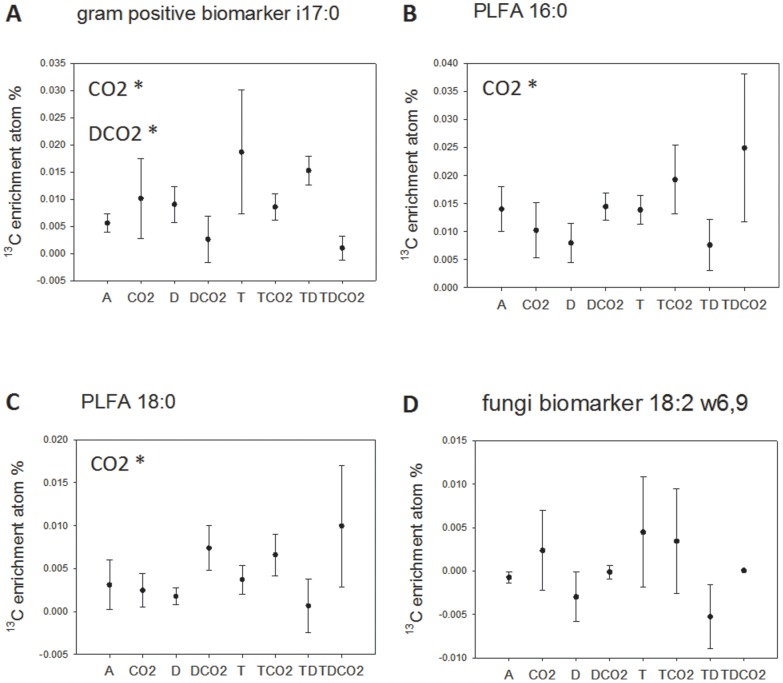
^13^C enrichment (atom%) in individual PLFAs. Gram positive biomarker *i*17:0 (**A**), the non-specific PLFA 16:0 (**B**), the non-specific 18:0 (**C**) and the fungi biomarker PLFA 18:2ω6,9 (**D**) one day after addition of ^13^C_2_-glycine to field plots subjected to *in situ* treatment through one year with the climate change factors: elevated atmospheric CO_2_ concentration (CO2), summer drought (D) and warming (T), and those treatments in all combinations. A is ambient control = no treatment. Error bars represent standard error. Significant effects of treatments: * is P<0.05.

^13^C enrichment of PLFAs after the *in situ* incubation with ^13^C-glycine suggested that the effects of climate treatment were significant. The largest ^13^C enrichment of biomarker PLFAs from the ^13^C-glycine label was found in the G^+^ biomarker *i*16:0. The main effect of elevated CO_2_ was significantly smaller ^13^C enrichment (*i.e.* microbial uptake) in: the G^+^ bacterial biomarker *i*17:0 (CO_2_: P = 0.0263; CO_2_*D: P = 0.0499, mixed model ANOVA with *Calluna* fine root biomass included as co-variate; Figure 3a) and larger ^13^C enrichment in the non-specific PLFAs 16:0 (CO_2_: P = 0.0422; Figure 3b), 16:1ω11c (CO_2_: P = 0.0298) and 18:0 (CO_2_: P = 0.0333 Figure 3c) and non-significant in the fungal biomarker 18:2ω6,9 (Figure 3d).

The specific microbial activity (τ; Equation III) was very different among individual biomarkers. The largest specific activity appeared in the non-specific biomarker 18:1ω9c with: τ = 151±94 and the bacterial biomarkers 15:0 with: τ = 33±9; and a15:0 with: τ = 15±4. Small specific activity of τ = 5 to 1 was observed in the bacterial biomarkers *i*15:0, 10Me16:0, *i*17:0, and 18:1ω9. Weak (τ<1) specific activity was observed in the fungal biomarker 18:2 ω6,9 and in 10Me17:0 and 16:0. There was no significant effect of treatment on τ.

## Discussion

### Experimental warming effects on soil microbes

The decrease in PLFA concentrations for bacteria and fungi in treatments with warming, reflects a change in the composition of the microbial community as found in parallel in a deciduous forest [65] and sub-arctic heath ecosystems [31]. Similar trends are frequent but not universal: In an old field ecosystem only the concentrations of fungal and G^−^ bacterial biomarkers decreased, while G^+^ bacteria increased in response to the warming treatment [60]. In laboratory incubations of soil from a boreal *Picea abies* forest, temperature oscillations around 0°C decreased bacterial PLFA concentration but increased fungal biomarkers [38]. These observed ecosystem specific responses furthermore appeared at different treatment response times, i.e. after treatment through one year in the case of the *in situ* treated temperate heathland (this study); after 6 months in the laboratory incubations of forest soil [38]; and, after more than 10 years at the sub-arctic heath ecosystem and in the deciduous forest [41], [65]. Storing of C in microbial membrane lipids, measured as soil PLFA concentrations, reflect the accumulated microbial response to the ecosystem manipulation treatments by: (i) quality: membrane lipid concentration can express physiological changes of the individual cell membranes which moderate the membrane fluidity (thickness and viscosity) to accommodate the environmental change [66], [67]; and (ii) quantity: change of the number of cells and the overall biomass due to bacterial growth based on changes in the available substrate. Inferred from this, the reduced concentration of fungal PLFAs in warming treatment suggests that a future warmer climate will lead to a reduced storage of C in soil microbes, whereby C may be sourced out of the soil as efflux in the long term.

### Bacteria and fungi use different C-substrates

Supplying an amino acid as an elicitor to activate the microbial community [68] and to trace the activity of microbes by ^13^C- enrichment of the PLFA biomarkers, provided an accurate measure of the microbial response to the climate change treatment and the specific microbial activity on a short term scale [52]. The ^13^C-enrichments and the high specific activity (τ) suggest that G^+^ bacteria expressed opportunistic substrate use, representing organisms with the strategy to quickly colonize new substrates and on a short term time scale incorporate most of the amended glycine substrate, compared to fungi often utilizing more recalcitrant organic matter (a parallel to ‘K’ and ‘r’ strategists, see [69]). Similar results were obtained after glucose addition to grassland soil [70]. There was no general direction in the response to the climate change treatments for incorporation of ^13^C from glycine across the analyzed biomarker PLFAs. However, the effects of elevated CO_2_ did suggest increased direct incorporation of glycine in microbial biomass, in particular in G^+^ bacteria, in an ecosystem subjected to elevated CO_2_. Conclusively, the treatment with elevated CO_2_ had mainly a stimulating effect on the bacterial usage of glycine on a short term scale. Hereby, elevated atmospheric CO_2_ may in the future increase the routing of C through bacteria, which may source an elevated efflux of CO_2_, as already observed in the experiment [51].

There is no universal effect of long-term treatment with elevated CO_2_ on microbial community structure across ecosystems, as some systems respond with increase in fungal biomass, and others with increase in bacteria. At a desert ecosystem a shift in microbial community structure was observed in the form of as decreased bacterial and increased fungal abundance [63]. By contrast, in one grassland the fungal to bacterial ratio increased [71], while for a different grassland a higher PLFA concentration of both bacteria and fungi was found [46]. At our temperate heathland site, we here found a large (G^+^) bacteria activity (τ) in response (glycine incorporation) to elevated CO_2_, while there was no effect of warming or drought. For fungi, the biomarker 18:2ω6,9 did not show large incorporation of the added ^13^C-glycine and the specific activity was very small (τ<1). Both suggest limited or no effective use of glycine by fungi in this soil. Interestingly, a contrasting conclusion was obtained at a ^13^C-CO_2_ pulse labeled grassland under elevated CO_2_ treatment [72], however, arbuscular mycorrhiza was the principal fungal group in contrast to the dominance of ericoid mycorrhizal *Calluna vulgaris* in our experiment. Irresponsiveness by the fungal biomarker 18:2ω6,9 to labile carbohydrate utilization was also seen with ^13^C incubation with labeled glucose, acetic acid, glycine, starch and vanillin in tundra soil [42], [12], and with glucose with grassland soil [30]. By contrast, the fungal biomarker 18:2ω6,9 had the highest depletion in ^13^C originating in the FACE (diff A to CO_2_ treatments 2.9‰). From this we infer that out of the investigated biomarkers the fungi probably had the largest usage of plant derived substrate, i.e. rhizodeposits or litter components. Hence, we conclude that the main function of the dominant fungi in these soils was saprotrophic litter decomposition, and not the use of fresh, labile carbohydrate substrates, suggesting there was no direct priming effect of the fungi activity, but instead the fungi represented important microbial components for long-term circulation of C in soil organic matter [52].

### Conclusions

The response to climate change treatments suggests that: i) bacterial usage of glycine will increase in ecosystems with elevated concentration of CO_2_ on a short-term time-scale. Hereby, elevated atmospheric CO_2_ may in the future increase the routing of C through bacteria, which may lead to an elevated efflux of soil CO_2_; and ii) at longer-term time-scale fungi will through plant litter decomposition regulate the slow circulation of C in SOM; iii) the observed general decrease in concentrations and diversity of PLFAs in response to warming (∼1°C) might indicate stress-dieback of minority types or altered cell membrane properties, although the intensity of warming was modest in our experiment.

All together, the bacterial (and not fungal) utilization of glycine in our experiment indicated substrate preference and resource partitioning within the microbial community. This thereby suggested a potential diversified response pattern between microbial groups to future changes in substrate availability and climatic factors. While plant root exudation of glycine might increase under elevated CO_2_, the resulting chemical composition of plant litter might decrease in quality for saprotrophic fungi. Herby, bacterial groups will advance their use of labile substrate (carbohydrates, amino acids), while saprotrophic fungi accessing recalcitrant substrate will rely on the poorer quality of SOM resource. Overall, we predict increased bacterial dominance in the soils of heathland ecosystems and a shift in functional decomposition of SOM at future climatic change.
